# A CD19-Anti-ErbB2 scFv Engager Protein Enables CD19-Specific CAR T Cells to Eradicate ErbB2^+^ Solid Cancer

**DOI:** 10.3390/cells12020248

**Published:** 2023-01-07

**Authors:** Andreas A. Hombach, Christine Ambrose, Roy Lobb, Paul Rennert, Hinrich Abken

**Affiliations:** 1 Department I Internal Medicine, University Hospital Cologne, Robert-Koch-Str. 21, 50931 Köln, Germany; 2 Center for Molecular Medicine Cologne, 50937 Cologne, Germany; 3 Aleta Biotherapeutics Inc., Natick, MA 01760, USA; 4 Leibniz Institute for Immunotherapy, Division Genetic Immunotherapy, University Regensburg, 93053 Regensburg, Germany

**Keywords:** chimeric antigen receptor, ErbB2 (Her2/neu), herceptin, scFv fusion protein, CD19

## Abstract

The efficacy of CD19-specific CAR T cells in the treatment of leukemia/lymphoma relies, at least in part, on the unique properties of the particular CAR and the presence of healthy B cells that enhance the target cell lysis and cytokine secretion through repetitive stimulation. Here, we report to apply the same CAR to target solid tumors, such as ErbB2^+^ carcinoma. CD19 CAR T cells are redirected towards the ErbB2^+^ cells by a fusion protein that is composed of the herceptin-derived anti-ErbB2 scFv 4D5 linked to the CD19 exodomain. The CD19-4D5scFv engager enabled CD19 CAR T cells to recognize the ErbB2^+^ cancer cells and to suppress the ErbB2^+^ tumor growth. The primary killing capacity by the ErbB2-redirected CD19 CAR T cells was as efficient as by the ErbB2 CAR T cells, however, adding CD19^+^ B cells furthermore reinforced the activation of the CD19 CAR T cells, thereby improving the anti-tumor activities. The ErbB2-redirected CD19 CAR T cells, moreover, showed a 100-fold superior selectivity in targeting cancer cells versus healthy fibroblasts, which was not the case for the ErbB2 CAR T cells. The data demonstrate that the CD19 CAR T cells can be high-jacked by a CD19-scFv engager protein to attack specifically solid cancer, thereby expanding their application beyond the B cell malignancies.

## 1. Introduction

The chimeric antigen receptors (CARs) with specificities for a broad panel of tumor associated antigens, were developed during the last decades, however, the best clinical responses are still obtained by the CD19-specific CAR T cells in the treatment of B cell malignancies [1]. This is attributed, at least in part, to the high CD19 levels on nearly all malignant B cells, along with those of healthy B cells that are likewise recognized by the CD19-specific CAR T cells. In addition, the intrinsic properties of the CD19 targeting domain FMC63 may also contribute to the success, such as the low tonic signaling capacities keeping CAR T cells in an activated state. In the treatment of solid cancer, moreover, the spectacular efficacy of the CAR T cell treatment could not be reproduced, raising the hypothesis whether the CD19^+^ healthy B cells can act as stimulators to boost the anti-leukemia/lymphoma activity of the CD19 CAR T cells. Redirecting the CD19 CAR T cells towards the solid cancer while also capable of engaging the endogenous healthy B cells would provide the benefit of the repetitive stimulation to the T cells.

Following our hypothesis, the CD19-specific CAR T cells need to be in close contact to the solid cancer cells in an antigen-mediated fashion. During the last years, the T cell engager proteins with dual or multiple specificities were developed, that bridge the CD3^+^ T cells to various cancer cells for inducing a T cell anti-cancer cell response [2]. As prototypes of such engager proteins, the bispecific antibodies (BsAbs) harbor two binding domains, one recognizing an antigen on the T cell, mostly the CD3, the other an antigen on the cancer cell surface. Such bi- and multi-specific antibodies in various formats have gained the broad potential in preclinical and clinical investigations, resulting in regulatory approvals for the treatment of hematologic malignancies. Following on the clinical success of the CD3e and CD19 engaging antibody blinatumomab [3], the bispecific antibodies targeting solid tumors were likewise pre-clinically and clinically explored, including targeting the CEA (carcinoembryonic antigen), ErbB2 (Her2/neu), PSMA (prostate-specific membrane antigen), GPC3 (glypican-3), and gpA33 (glycoprotein A33) [2,4,5,6,7]. While most target proteins on solid cancer cells are also expressed by healthy cells, such as ErbB2, the binding domain was re-engineered in favor of a low affinity to reduce the binding to the low antigen expressing healthy cells and finally to reduce the on-target off-tumor toxicity [8]. By changing the valence of the antibodies, delivering the bispecific antibodies in the form of prodrugs, or identifying new targets, it is currently aimed to improve the T cell activation towards solid tumors. In addition, the bispecific antibodies redirecting the T cells towards the antigen-presenting cells and augmenting their activation by enhancing the B7/CD28 co-stimulation, may allow for establishing a more durable anti-cancer immune response [9]. Accordingly, a tri-specific antibody that targets the ErbB2, CD3, and CD28, induces the regression of breast cancers through a mechanism involving the CD4-dependent inhibition of the cell cycle progression of the cancer cells [10], moreover highlighting the supportive impact of the CD4^+^ T cells, in addition to the cytotoxic CD8^+^ T cells. In this line, a combination of techniques is likely required, to achieve a more pronounced T cell response against the solid tumors.

We hypothesized to produce a powerful immune response against solid tumors by combining the antibody redirected cancer cell targeting, the CD28-CD3ζ triggered T cell activation through a CAR, and the repetitive restimulation of the retargeted T cells by engaging the endogenous B cells. We here exemplarily explored the retargeting of the CD19-specific CAR T cells towards the ErbB2^+^ cancer cells, while mainly sparing the ErbB2^+^ healthy fibroblasts, through a fusion protein that consists of the ErbB2 targeting single chain variable fragment (scFv) antibody 4D5, fused to the extracellular domain of CD19 that is recognized by the CAR delivering the CD28 and CD3ζ stimulation to the engineered T cell upon binding [2]. We here demonstrate that the CD19-4D5scFv fusion protein redirects the CD19-specific CAR T cells towards the ErbB2^+^ cancer cells and activates the CAR T cells to efficiently eliminate the CD19^−^ ErbB2^+^ cancer cells in vitro and in a xenograft mouse model. While the herceptin-derived anti-ErbB2 CAR produced serious side effects, so far hampering its further clinical exploration [3], the targeting selectivity of the fusion protein redirected the CAR T cells towards the cancer cells versus the healthy fibroblasts with the physiological ErbB2 levels, was unexpectedly high. Data exemplarily demonstrate that the CD19-specific CAR T cells can be redirected by a CD19 engager protein towards the solid cancer cells while being additionally triggered by the presence of the B cells through the CAR for the augmented killing capacities, suggesting their exploration in the treatment of solid cancer.

## 2. Materials and Methods

### 2.1. Cell Lines and Reagents

HEK293T cells are human embryonic kidney cells that express the SV40 large T antigen (kindly provided by R. Bolhuis, Erasmus MC, Rotterdam, NL) [11]. SKOV3 (ATCC HTB-77) ovarian cancer and LS 174T (ATCC CL-188) colorectal cancer cell lines were obtained from ATCC, Rockville, MD, USA. The MC38 cells are mouse fibrosarcoma cells (kindly provided by Dr. M. Neumaier, UMM, Mannheim, Germany). Human dermal fibroblasts were purchased from tebu-bio, Offenbach, Germany. The engineering of the CD19-expressing HEK293T cells was described earlier [12]. All cell lines were cultured in RPMI 1640 or a DME medium, 10% (*v*/*v*) fetal bovine serum (FBS) (ThermoFisher Scientific, Erlangen, Germany). Anti-CD3 monoclonal antibody (mAb) OKT3 and anti-CD28 mAb 15E8 were purified by affinity chromatography from the OKT3 (ATCC CRL 8001) and 15E8 hybridoma (kindly provided by Dr. R. van Lier, Red Cross Central Blood Bank, Amsterdam, The Netherlands) supernatants, respectively. The PE- and FITC conjugated F(ab’)2 goat anti-human IgG antibodies were purchased from Southern Biotechnology, Birmingham, AL, USA. The fluorophore-conjugated anti-CD3, anti-CD20 and anti-CD19 mAbs were purchased from Miltenyi Biotec, Bergisch Gladbach, Germany.

### 2.2. Preparation of the Human T Cells

Peripheral blood lymphocytes were obtained from healthy donors by Ficoll density centrifugation. The T cells were activated by incubation with OKT3 and 15E8 mAbs (100 ng/mL each) and IL-2 (500 U/mL) and further propagated in the presence of IL-2 (500 U/mL). Blood was obtained from healthy volunteers through the University Hospital, Dept. of Transfusion Medicine (ethic approval 21-2224-101).

### 2.3. CAR T Cell Generation

The generation of the CD19-, CEA- and ErbB2 CARs with the FMC63, BW431/26 and 4D5 scFv binding domains, respectively, were previously described in detail [13,14,15]. Retroviruses were produced by the HEK293T cells upon co-transfection of plasmid DNAs encoding GALV, gag/pol, and the CAR, respectively, as described [16,17]. The T cells were stimulated as described above and transduced with γ-retrovirus containing supernatants of transfected HEK293T cells.

### 2.4. Generation and Expression of the CD19-Fusion Proteins

The generation, expression and purification of the CD19-4D5scFv (#42), CD19-his tagged (#28) and scFv-Fc fusion proteins was described recently in detail [18,19]. The 4D5scFv-Fc fusion protein was transiently expressed by the transfected HEK293T and secreted into the supernatant.

### 2.5. Flow Cytometry and Cell Sorting

The CAR T cells were stained with fluorophore-labeled antibodies and the cells were analyzed by a FACSCanto II flow cytometer equipped with the FACSDiva software (BD Bioscience). The healthy B cells from the peripheral blood were flow sorted with a FACS Aria using a fluorophore-labeled anti-CD20 mAb.

### 2.6. Antigen Specific CAR T Cell Activation

CAR T cells (0.125–10 × 10^4^ cells/well) were co-cultivated for 24–48 h in 96-well round bottom plates with target cells (each 2 × 10^4^ cells/well) in the absence or presence of #42 and #28 fusion proteins (0.001–1000 ng/mL). The specific cytotoxicity of the CAR T cells was monitored by a XTT based colorimetric assay [20] using the “Cell Proliferation Kit II” (Roche Diagnostics, Mannheim, Germany). The test viability of the tumor cells was calculated as mean values of six wells containing only tumor cells subtracted by the mean background level of the wells containing the medium only. The mean values of the non-specific formation of formazane due to the presence of the T cells was determined from the triplicates containing T cells in the same number as in the corresponding experimental wells. The number of viable tumor cells in the experimental wells was calculated as follows: viability (%) = [OD(experimental wells-corresponding number of T cells)]/[OD(tumor cells without T cells-medium)] × 100. Cytotoxicity (%) was defined as 100-viability (%).

### 2.7. ELISA

IFN-γ in culture supernatants was monitored by ELISA. Briefly, ELISA plates were coated overnight at 4 °C with the anti-IFN-γ capture antibody (1 µg/mL) and blocked with 3% BSA at room temperature. The supernatants were incubated for 2 h at room temperature and bound IFN-γ was detected by the biotinylated anti-IFN-γ detection antibody (0.5 µg/mL). ScFv-Fc fusion proteins in supernatants were quantified by ELISA utilizing anti-human IgG1 capture (1 µg/mL) and biotinylated detection (0.5 µg/mL) antibodies, respectively. The reaction product was visualized by a peroxidase-streptavidin conjugate (1:10,000) and ABTS (Roche Diagnostics).

### 2.8. In Vivo Suppression of the SKOV3 Tumors by the CD19-4D5scFv Redirected Anti-CD19 CAR T Cells

Rag2^−/−^ cγ^−/−^ mice (Charles River, Sulzfeld, Germany) (five mice/group) were subcutaneously grafted with ErbB2^+^ SKOV tumor cells (5 × 10^6^ cells/mouse). All mice established tumors and were treated i. v. twice with two doses of #42 or #28 fusion proteins (200 µg/mouse) and CD19scFv-CAR T cells (3 × 10^5^ cells/mouse and 5 × 10^5^ cells/mouse, respectively) and once with fusion proteins, only, at indicated time points. The tumor volumes were recorded every 2–3 days by digital caliper measurements. The mean values (A) and area under curve (AUC) (B) were determined, as previously described [21] and the statistics were determined by the Student’s *t* test. The mouse experiments were carried out under the approval of the local animal welfare board.

### 2.9. Statistics

The experimental results from the independent representative experiments are reported as mean values ± standard deviation (SD). Significance analyses were performed by the two-sided Student’s *t* test using Microsoft Excel.

## 3. Results

**Figure 1:**
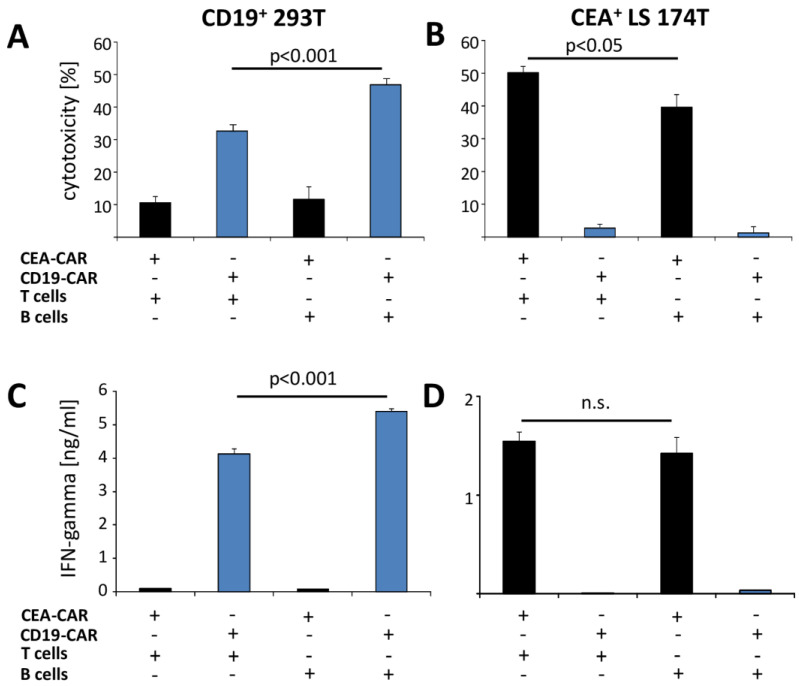
Pre-incubation with the autologous B cells improves a CD19-CAR T cell attack against CD19^+^ target cells. Autologous B and T cells were flow sorted utilizing anti-CD20 and anti-CD3 mAbs, respectively, and co-incubated (1:10) with CD19-CAR T cells (blue columns) for 72 h; CEA-CAR T cells served as control. CAR T cells were recovered and co-incubated (5 × 10^4^ cells/well) for 48 h with engineered CD19^+^ CEA^−^ HEK293T (**A**,**C**) or CD19^−^ CEA^+^ LS 174T (**B**,**D**) cells as targets for the respective CAR (each 2.5 × 10^4^ cells/well), respectively. (**A**,**B**) Viability of the tumor cells was determined by the XTT assay and specific cytotoxicity was calculated. (**C**,**D**) IFN-γ in the culture supernatant was recorded by ELISA. Data represent mean values of triplicates ± SD. Significant differences were determined by Student’s *t* test; n.s. = not significant.

We asked whether the CD19-CAR T cells can be efficiently redirected against the ErbB2^+^ cancer cells while taking advantage of the beneficial properties of the CD19-CAR [22]. Moreover, we expected a beneficial impact on the CD19 CAR T cells upon the repetitive stimulation through the healthy B cells. To address this issue, we engineered human T cells from the peripheral blood with the CD19-CAR harboring the FMC63 scFv targeting domain and the intracellular CD28-CD3ζ signaling domains [13]. The CD19-CAR T cells were incubated with autologous B cells prior to adding to the CD19^+^ target cells. As expected, the CD19-CAR T cells killed the CD19 engineered HEK293T target cells, whereas the carcinoembryonic antigen (CEA) specific CAR T cells did not (Figure 1A). Notably, the pre-incubation with the autologous CD19^+^ healthy B cells, but not with T cells from the same donor and in same numbers, significantly improved the subsequent killing of the CD19^+^ tumor cells (Figure 1A), whereas the killing of the CEA^+^ colorectal cancer cells by the CEA-CAR T cells for comparison, was unaffected (Figure 1B). We concluded that pre-stimulation with B cells enhanced the killing capacity of CD19-CAR T cells against the CD19^+^ target cells. Our conclusion was confirmed by the increased IFN-γ release of the CD19-CAR T cells pre-stimulated with healthy B cells, that was not observed for the CAR T cells with an irrelevant specificity (Figure 1C,D).

**Figure 2:**
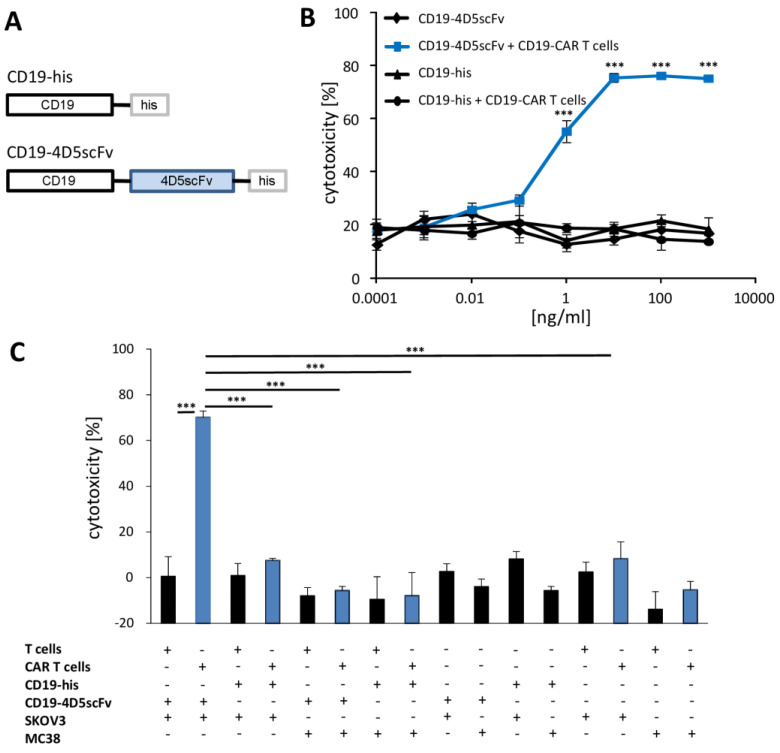
The fusion protein CD19-4D5scFv redirects the CD19-CAR T cells to kill specifically the ErbB2^+^ SKOV-3 tumor cells. (**A**) Schematic presentation of the fusion proteins consisting of the extracellular CD19 domain, with or without the anti-ErbB2 4D5scFv, and the linked his tag. (**B**) CD19-CAR T cells (blue line) or non-modified T cells (2 × 10^4^ cells/well) were co-incubated with ErbB2^+^ human SKOV-3 cells (2 × 10^4^ cells/well) in presence of serial dilutions of the CD19-4D5scFv or CD19-his fusion protein (0.0001–1000 ng/mL) for 18 h. (**C**) CD19-CAR T cells (blue columns) were co-incubated (2 × 10^4^ cells/well) for 18 h with human ErbB2^+^ SKOV-3 or mouse ErbB2^+^ MC38 tumor cells for the control (each 2 × 10^4^ cells/well) in presence of the CD19-his- or CD19-4D5scFv fusion protein (each 10 ng/mL). For the control, the assay was performed with non-transduced T cells, CD19-CAR T cells in absence of a fusion protein, and in the presence of the fusion proteins without T cells, respectively. Viability was determined by the XTT assay and the specific cytotoxicity was calculated. Values represent the mean of the technical replicates ± SD. Significant differences were calculated by Student’s *t* test: *** *p* < 0.001.

To redirect the CD19-CAR T cells towards the ErbB2^+^ cancer cells, we used the CD19-4D5scFv fusion protein (#42) consisting of the extracellular CD19 domain and the herceptin-derived 4D5scFv binding domain linked to a his tag, as previously reported [18] (Figure 2A); the CD19-his tagged protein (#28) served as the control. The binding properties of the respective proteins were demonstrated elsewhere [18]. We redirected the CD19-CAR T cells against the ErbB2^+^ SKOV-3 ovarian cancer cells by adding the CD19-4D5scFv fusion protein. As summarized in Figure 2B, the CD19scFv CAR T cells killed the ErbB2^+^ SKOV-3 cells dependent on the dose of the CD19-4D5scFv fusion protein with a half-maximal effective dose (ED50) of about 1 ng/mL protein; the maximum killing was achieved at concentrations >10 ng/mL. The CD19-4D5scFv fusion protein itself in concentrations of as high as 1000 ng/mL, did not produce any cytotoxicity towards the SKOV-3 cells. While in presence of the CD19-4D5scFv fusion protein, the CD19-CAR T cells recognized and killed the SKOV-3 cells; this was not the case in the presence of the CD19 protein without targeting domain or without added protein (Figure 2C). The killing of the ErbB2^+^ cells was specific, since the murine ErbB2^+^ MC38 cancer cells were not eliminated. We concluded that the CD19-4D5scFv fusion protein mediated the recognition of the human ErbB2^+^ cancer cells and finally their elimination by the activated CD19-CAR T cells.

**Figure 3:**
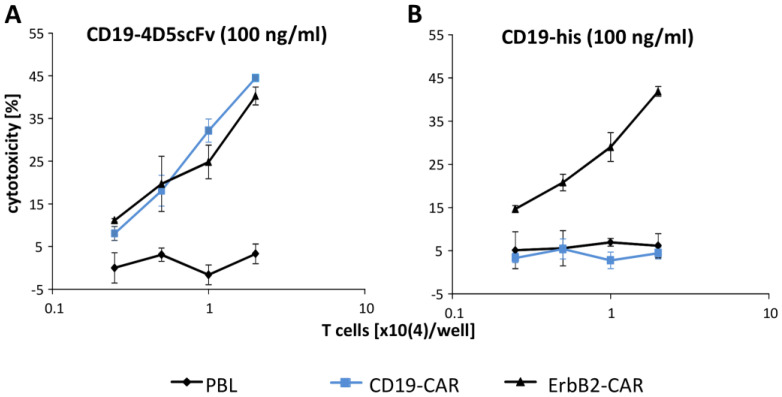
CD19-4D5scFv redirected the CD19-CAR T cells to kill the ErbB2^+^ tumor cells with the same efficiency as the ErbB2-CAR T cells. T cells were engineered with the CD19-CAR (blue lines) or ErbB2-CAR and the same numbers of CAR T cells (0.25–2 × 10^4^ cells/well) were co-incubated for 18 h with the ErbB2^+^ SKOV-3 tumor cells (2 × 10^4^ cells/well) in the presence of the CD19-4D5scFv (**A**) or CD19 fusion protein (**B**) for the control (100 ng/mL each). Non-transduced T cells also served as the control. Viability was determined by the XTT assay and the specific lysis was calculated. Values represent the mean of the triplicates ± SD.

We compared the efficiency in targeting the ErbB2^+^ cancer cells by the CD19-4D5scFv redirected CD19-CAR T cells with those by the ErbB2-CAR T cells. The ErbB2-CAR and the CD19-4D5scFv engager protein harbored the same ErbB2 targeting domain 4D5. The T cells engineered with the CD19-CAR or the 4D5scFv-CAR were incubated with the SKOV-3 cells in the presence of the CD19-4D5scFv, or CD19 protein without targeting the domain for the control, at saturating concentrations for the maximum killing. In the presence of 100 ng/mL of the CD19-4D5scFv fusion protein, the CD19-CAR T cells eliminated the SKOV-3 cells as efficiently as the 4D5scFv-CAR T cells at the same effector-to-target (E:T) ratio (Figure 3A). In contrast, the CD19 control protein did not induce the killing of the SKOV-3 cancer cells by the CD19-CAR T cells.

**Figure 4:**
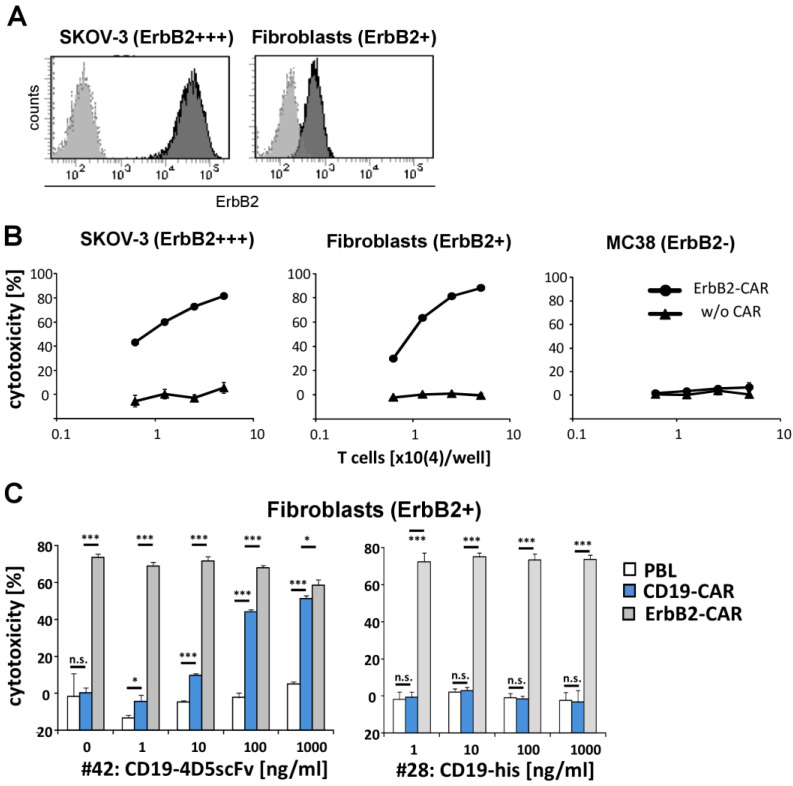
CD19-4D5scFv redirected CD19-CAR T cells discriminate between healthy ErbB2^low^ fibroblasts and the ErbB2^high^ SKOV-3 cancer cells while the 4D5scFv-CAR T cells do not. (**A**) Binding of the 4D5scFv to SKOV-3 cancer cells and healthy fibroblasts. Cells were incubated with 100 ng/mL of the 4D5scFv-Fc fusion protein or human IgG1 for the control, for 30 min at 4 °C. Bound antibodies were detected by an anti-human IgG-PE antibody, analyzed by flow cytometry and histograms were overlayed. (**B**) Killing of the SKOV-3 cancer cells and healthy fibroblasts by the anti-ErbB2 CAR T cells. The T cells were engineered with ErbB2-CAR and co-incubated (0.625–5 × 10^4^ cells/well) for 48 h with human SKOV-3 cells, healthy human fibroblasts, and murine ErbB2-MC38 cancer cells (each 2 × 10^4^ cells/well), respectively. Non-transduced T cells served as controls. Viability was determined by the XTT assay and the specific lysis was calculated. Values represent the mean of the technical replicates ± SD. (**C**) Different killing of the healthy fibroblasts by the CD19-4D5scFv redirected CD19-CAR T cells and the ErbB2-CAR T cells. The T cells were engineered with anti-CD19 and anti-ErbB2 CARs, respectively, and co-incubated (2 × 10^4^ cells/well) for 18 h with human fibroblasts (2 × 10^4^ cells/well), in the presence of serial dilutions (1–1000 ng/mL) of CD19-4D5scFv (#42) and CD19-his (#28) fusion protein, respectively. For the controls, non-transduced T cells and CD19 fusion protein without ErbB2 binding domain were used. Viability was determined by the XTT assay and the specific cytotoxicity was calculated. Values represent the mean of the technical replicates ± SD. Significant differences were determined by Student’s *t* test: *** *p* < 0.001; * *p* < 0.05; n.s. = not significant.

The clinical application of the ErbB2 specific CAR T cells with the herceptin derived 4D5 scFv binding domain, produced fatal side effects [23], most likely due to the off-tumor on-target activation of the CAR T cells against the healthy tissues with physiological ErbB2 levels. Accordingly, the ErbB2-CAR T cells with the 4D5scFv killed the ErbB2^low^ fibroblasts from the healthy donors with nearly the same efficiency as the SKOV-3 cancer cells with high ErbB2 levels (Figure 4A,B). The killing of human fibroblasts was specific since the MC38 cells with murine ErbB2 were not killed.

We asked whether the CD19-4D5scFv redirected CD19-CAR T cells show a superior safety profile than the 4D5scFv-CAR T cells, with respect to discriminating between the ErbB2^low^ healthy cells and the ErbB2^high^ cancer cells. To address this issue, we co-incubated T cells with CD19-CAR and 4D5-CAR, respectively, with human fibroblasts in the presence of serial dilutions of the CD19-4D5scFv fusion protein or the CD19 control protein. As summarized in Figure 4C, the 4D5-CAR T cells killed the healthy human fibroblasts with a high efficiency, as they killed the SKOV-3 cells. In contrast, the CD19-4D5scFv redirected CD19scFv-CAR T cells kill the healthy fibroblasts only in presence of more than 100 ng/mL fusion protein, which is 100-fold higher than the ED50 for ErbB2 cancer cell killing. The toxicity was protein dose dependent and declined to the background below the 10 ng/mL CD19-4D5scFv fusion protein that was still sufficient for maximum killing of the SKOV-3 cancer cells (cf. Figure 2). We did not observe any toxicity of the CD19-CAR T cells against the fibroblasts in the presence of the CD19 control protein, furthermore, demonstrating the specificity in the redirected targeting (Figure 4C).

**Figure 5:**
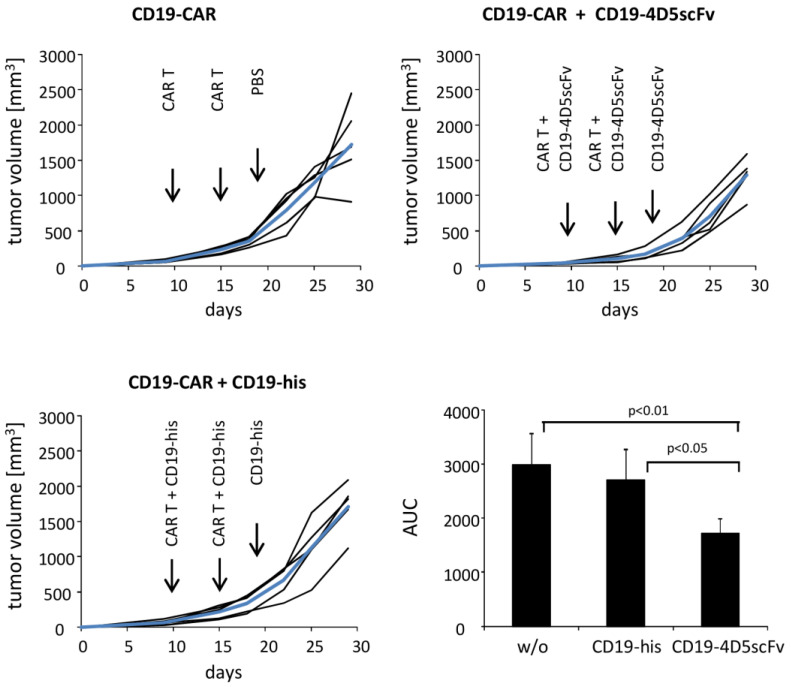
CD19-4D5scFv redirected CD19-CAR T cells suppressed the growth of the ErbB2^+^ SKOV-3 tumors. Rag2^−/−^cγ^−/−^ mice (five mice/group) were subcutaneously inoculated with the ErbB2^+^ SKOV-3 tumor cells (5 × 10^6^ cells/mouse). Mice with established tumors were i.v. treated with the CD19-CAR T cells on day 9 (3 × 10^5^ cells/mouse) and day 15 (5 × 10^5^ cells/mouse). Fusion protein CD19-4D5scFv or CD19-his (each 200 µg/mouse) were co-injected, as indicated; on day 18 fusion protein without CAR T cells was injected, PBS served as the control. Tumor growth was recorded and the tumor volumes were determined (mean values: bold blue lines). Areas under curves (AUC) were calculated, mean values were determined, and significant differences were determined by Student’s *t* test.

We addressed whether the CD19-CAR T cells can be redirected against the established ErbB2^+^ solid tumors in vivo, by adding the CD19-4D5scFv engager protein. To address this issue we grafted Rag2^−/−^cγ^−/−^ mice (5 mice/group) subcutaneously with the SKOV-3 cancer cells. The mice with established tumors (tumor size >50 mm^3^) were treated twice by intravenous injections of CD19-CAR T cells; the CD19-4D5scFv or CD19-his fusion protein was injected at day 9, day 15, and day 18 (each 200 µg per mouse). As summarized in Figure 5, the CD19-CAR T cells significantly inhibited the tumor growth upon application of the CD19-4D5scFv fusion protein, compared to the CD19-CAR T cells without the fusion protein or in the presence of the CD19-his protein for the control.

Taken together, the CD19-CAR T cells can be efficiently redirected by the CD19-4D5scFv protein against the ErbB2^high^ cancer cells in vitro and in vivo, without attacking the human fibroblasts with the physiological ErbB2 levels.

## 4. Discussion

The lasting efficacy of the CD19-CAR T cell therapy in the treatment of leukemia/lymphoma is thought to be partly due to the repetitive co-stimulation by the engagement of healthy B cells [24,25]. In line with this assumption, we recorded improved killing of the CD19^+^ target cells upon pre-incubating the CD19-CAR T cells with autologous healthy B cells; the effect cannot be achieved by adding autologous T cells. These data, along with the reported low tonic signaling capacity, provided us the rationale to redirect a CD19-CAR T cell attack against the CD19-negative target cells of solid tumors.

We here engrafted the additional specificity to the CD19scFv-CAR T cells by adding a composite protein consisting of the herceptin derived anti-ErbB2 4D5scFv domain for the cancer cell targeting and of the CD19 ectodomain, to allow the engagement by the anti-CD19 CAR. Thereby, the CD19-CAR T cells gain specificity for the ErbB2^+^ cancer cells. The data clearly indicate the newly gained specificity of the CD19-CAR T cells for ErbB2 in the presence of the fusion protein; levels of 1 ng/mL fusion protein are sufficient to induce the half-maximal killing capacities of the CD19-CAR T cells towards the ErbB2^+^ target cells in vitro, a concentration that can likely be achieved in vivo. Accordingly, the injection of the fusion protein at 5-day-intervals into the mouse redirected CD19-CAR T cells to attack the ErbB2^+^ CD19^−^ tumors; injections of the CD19 protein without targeting the domain had no effect.

ErbB2 is also expressed by healthy epithelia, although at lower densities than by cancer cells. This situation can produce fatal side effects, as reported for the CAR T cells with the herceptin derived 4D5scFv binding domain [23]. In line with these observations, we recorded the cytolytic activation of the 4D5scFv-CAR T cells against healthy fibroblasts with physiological ErbB2 levels. While the CD19-CAR T cells retargeted by the CD19-4D5scFv protein lysed ErbB2^+^ cancer cells with similar efficacy as the ErbB2-CAR T cells, we found a substantially lower toxicity against the healthy fibroblasts at the CD19-4D5scFv protein doses that were still effective for mediating the ErbB2^+^ cancer cell killing. In particular, the efficient dose for the cancer cell recognition was 100-fold lower than for the recognition of healthy fibroblasts in a CD19-CAR T cell in vitro assay. In case of any toxicity, the CD19-4D5scFv protein is expected to be rapidly cleared from circulation, limiting the toxicity in time. That is not the case for canonical ErbB2-CAR T cells. Moreover, the cytokine release syndrome (CRS) and neurotoxicity are potentially life-threatening side effects of the CD19-CAR T cells in a subset of patients. These side effects need vigilant attention but are manageable, generally reversible, and may contribute also to the therapeutic benefit of the CD19 CAR T cells [26].

Taken together, we exemplarily demonstrate that the CD19-CAR T cells can be specifically redirected by a modularly composed scFv-CD19 fusion protein to muster an efficient CD19-CAR T cell attack against the CD19-negative cancer cells. By using the fusion proteins of this and other specificities, the strategy will broaden the application of the well-established CD19-CAR T cell therapy, towards solid cancer and other targetable organs.

## Data Availability

Data are available from the authors on request.
